# Molecular mechanisms of anti-tumor properties of P276-00 in head and neck squamous cell carcinoma

**DOI:** 10.1186/1479-5876-11-42

**Published:** 2013-02-18

**Authors:** Prabha B Mishra, Aurelio S Lobo, Kalpana S Joshi, Maggie J Rathos, Gopinath A Kumar, Muralidhara Padigaru

**Affiliations:** 1 Biomarker Discovery Group, Department of Pharmacology, Piramal Healthcare Ltd, 1-Nirlon Complex, Goregaon (East), Mumbai, Maharashtra, 400063, India; 2 Target Identification group, Piramal Healthcare Ltd, Mumbai, Maharashtra, 400063, India; 3 Department of Pharmacology, Piramal Healthcare Ltd, Mumbai, Maharashtra, 400063, India; 4 School of Bio Sciences and Technology, VIT University, Vellore, Tamil Nadu, 632014, India

**Keywords:** FaDu, P276-00, Cyclin dependent kinase, Cyclin D, E2F1, HNSCC

## Abstract

**Background:**

Tumors of the head and neck present aggressive pathological behavior in patients due to high expression of CDK/CCND1 proteins. P276-00, a novel CDK inhibitor currently being tested in clinic, inhibits growth of several cancers *in vitro* and *in vivo*. The pre clinical activity of P276-00 in head and neck cancer and its potential mechanisms of action at molecular level are the focus of the current studies.

**Method:**

We have investigated the anti-cancer activity of P276-00 in head and neck tumors *in vitro* and *in vivo*. Candidate gene expression profiling and cell based proteomic approaches were taken to understand the pathways affected by P276-00 treatment.

**Results:**

It was observed that P276-00 is cytotoxic across various HNSCC cell lines with an IC_50_ ranging from 1.0-1.5 μmoles/L and culminated in significant cell-cycle arrest in G1/S phase followed by apoptosis. P276-00 treatment suppressed cell proliferation through inhibition of CCND1 expression, reduced phosphorylation of retinoblastoma protein and abrogative transcription of E2F1 gene targets. Further, we observed that apoptosis was mediated through P53 activation leading to higher BAX/BCL-2 ratio and cleaved caspase-3 levels. It was also seen that P276-00 treatment reduced expression of tumor micro-environment proteins such as IL-6, secreted EGFR and HSPA8. Finally, P276-00 treatment resulted in significant tumor growth inhibition in xenograft tumor models via lowered proliferative activity of E2F1 and aggravated P53 mediated apoptosis.

**Conclusion:**

In summary, we have observed that P276-00 inhibits cyclin-D/CDK4/P16/pRB/E2F axis and induces apoptosis by increased P53 phosphorylation in HNSCC cells. These results suggest a novel indication for P276-00 in head and neck cancer with a potential role for IL-6 and HSPA8 as candidate serum biomarkers.

## Background

Head and neck squamous cell carcinoma (HNSCC) is the 8^th^ most common cause of morbidity and mortality due to cancer worldwide. Head and neck cancer refers to carcinoma arising in the mucosal surfaces of the oral cavity, oropharynx, larynx and hypopharynx of which 90% are HNSCC [1]. Current treatment options for HNSCC include surgery, γ-irradiation, and chemotherapy depending on the site, size and the stage of lesions. Aberrant expression of P14, P16, P53, CCND1, RAS, EGFR, and MYC is known to initiate tumorigenesis and contribute towards development of HNSCCs [2]. It has also been observed that tumor cells have deregulated CDK or CCND1 activity or combination of both [3]. Abnormalities in the expression of a cell cycle regulatory protein such as CCND1 due to genomic inversions, translocation and gene amplification have been identified as a cause for poor prognosis and drug resistance in HNSCC. Amplification of CCND1 has been observed in 58% of cases with over expression of protein seen in 68% of HNSCC; [4,5]. CCND1 plays a central role in cell cycle progression by promoting G1/S cell cycle transition. CCND1 binds cyclin-dependent kinase 4 or 6 (CDK4/6) to form an active kinase that induces phosphorylation of retinoblastoma protein (RB) resulting in release of E2F transcription factors and thereby promoting transcription of genes required for cell cycle progression [6-8]. Further, inactivation of the P53 pathway is a salient feature of molecular mechanism of HNSCC progression [9]. P53 mutations are associated with drug resistance and treatment failure and determine the overall survival of HNSCC patients [10,11]. Tumor microenvironment plays a critical role in initiation and progression of tumor and includes the extracellular matrix proteins, cytokines, proteases and hormones [12]. IL-6, EGFR and HSPA8 act as survival factors for neoplasm and increased circulating levels of these proteins have been observed in various types of cancer patients [12-14].

Therapeutic agents such as Erbitux (EGFR inhibitor), Bevacizumab (VEGF inhibitor) and Erlotinib (EGFR inhibitor) that target cellular signaling pathways have been developed as promising candidates to treat HNSCC [12]. At the same time, small molecules which are acting as competitors of ATP binding with CDK are of particular relevance in designing therapy for HNSCC.

P276-00 is a flavone with cyclin-dependent kinase inhibitor activity and arrests the cells in G1/S phase transition [15,16]. Kinase assay profiling studies have shown that P276-00 selectively inhibits CDK4-CCND1 complex as compared to CDK2-CCNE1 with a 40-fold specificity. P276-00 is also observed to inhibit CDK9-CCNT1 mediated RNA polymerase II-dependent transcription [17]. In the current study, we have investigated the activity of P276-00 in HNSCC and explored the potential mechanism of action of anti-cancer activity in multiple HNSCC cancer cells using *in vitro* and *in vivo* models.

## Methods

### Antibodies

Following primary antibodies were used for various analyses: CCND1, CCNE1, Phospho-RB^Ser780^ (pRB) and E2F1 (Santa Cruz biotechnology); PCNA (Chemicon); KI67(BD Pharmigen); cleaved-Caspase-3, phospho-P53^Ser392^ and P16 (Epitomics); β-Actin antibody (Sigma).

### Cell lines and culture conditions

Human squamous cell carcinoma cell lines- FaDu, Detroit-562 and SCC-25 were obtained from the American Type Culture Collection (Manassas, VA). FaDu and Detroit-562 cells were grown in EMEM medium and SCC-25 cells were grown in 1:1 mixture of DMEM and Ham’s F12 medium. Both the mediums were supplemented with 10% FBS (Hyclone) and 100 U/ml penicillin (Sigma).

### Tumor xenograft model

All animal experiments were handled in accordance with the guidelines of “Committee for the Purpose of Control and Supervision of Experiments on Animals” which was accredited by the Institutional Animal Ethics Committee of Piramal Healthcare. FaDu cells were harvested and re-suspended in saline at 10 million cells/0.2 mL volume and injected to severe combined immunodeficient (SCID) mice on right flank. Animals were randomized when tumor size attained a diameter of ~5 mm into control and treatment group. Treatment group received 50 mg/kg P276-00 solution in water by intraperitoneal route for 18 days. Tumor diameters were measured every alternate day and tumor growth inhibition (GI) was calculated as previously reported [15,16].

### Cell viability assay

The cytotoxicity of P276-00 was determined using the cell counting kit-8 (CCK8) as per the manufacturer’s instructions (Dojindo, Gaithersburg, MD, USA). The change in color due to assay was measured at 450 nM using a Tecan sapphire multi-fluorescence microplate absorbance reader (Tecan, Germany).

### Cell cycle analysis

Treated and untreated control cells were fixed in 70% ethanol and stained with Propidium iodide (50 mg/ml) and RNase A (1 mg/ml). DNA content was measured by flow cytometer (FACS Calibur Becton Dickinson, San Jose, CA). Cells with DNA content between 2 N and 4 N were designated as being in G1, S, and G2-M phases of the cell cycle. Cells exhibiting <2 N DNA content were designated as sub-G0 cells. The number of cells in each cell cycle compartment was expressed as a percentage of the total number of cells.

### mRNA expression assay

Extraction of total RNA was carried out using chloroform-ethanol precipitation method. Cells/tissues were lysed using Trizol Reagent (Invitrogen Corporation, Carlsbad, USA). The RNA was purified using RNeasy Mini kit (Qiagen GmbH, Hilden, Germany). For cDNA conversion, 2 μg of total RNA was reverse transcribed to cDNA using 200 units of Superscript III (Invitrogen Corporation, Carlsbad, CA, USA) as per manufacturer’s protocol. Further real-time PCR was performed in 96-well plates using Realplex™ Mastercycler System (Eppendorf, Germany) and the fluorescent SYBR green dye (Quantifast™ SYBR Green PCR Kit., Qiagen GmbH, Hilden, Germany). Data were analyzed using the Realplex™ software (version 1.5). The relative expression of genes was calculated with the relative Ct method [18] using GAPDH as housekeeping gene for normalization of data.

### Western blotting

FaDu cells were lysed with cell lysis buffer (cell signaling), resolved on a 10% SDS-PAGE gels and transferred to nitrocellulose membrane (Sigma-Aldrich, MO, USA). Membrane was blocked with 5% non fat milk (Santa Cruz Biotechnology, city, CA, USA) and treated with the primary antibody overnight at 4°C followed by HRP conjugated secondary antibody. After incubation, bound antibodies were detected with enhanced chemifluorescence (ECF) substrate (Sigma-Aldrich, MO, USA) and analyzed by Kodak Image station 4000MM (Kodak, Roches, CA).

### Cell based automated fluorescence imaging

FaDu cells were grown in 96-well plates and treated with different concentrations of P276-00 for 24 hours. Treated/untreated cells were fixed with 3.7% formaldehyde (Merck Biosciences, Darmstadt, Germany) for 20 min. and permeabilized using chilled permeabilization buffer (0.15% Triton X-100; Thermo Scientific, Waltham, MA) for 1.5 min. Subsequently the cells were blocked with DPBS containing 5% FBS and 5% goat serum (Vector Laboratories Inc., Burlingame, CA) for 1 hour at room temperature. Thereafter cells were incubated with primary antibody at 4°C overnight. This was followed by staining with DyLight™ 549-conjugated goat antibody (Thermo Scientific; Waltham, MA) and nuclear staining with Hoechst 33342 (AnaSpec Inc; Fermont, CA). The plates were scanned to acquire images on the ArrayScan® HCS reader (Thermo Scientific.). All the data points were analyzed using compartmental analysis bio-algorithm (Cellomics Inc. Pittsburgh PA). The results were expressed as percentage (%) deregulation as compared to control (vehicle) cells for each protein. The 50% effective concentrations (EC_50_) were calculated using nonlinear regression method with GraphPad software (Prism 5).

### Elisa

Supernatants were collected from treated and untreated cells and assayed for HSPA8 and IL-6 by ELISA (Enzyme-Linked Immunosorbent Assay) using HSPA8 ELISA kit (USCN Life Sciences Inc, Wuhan, China) and IL-6 ELISA kit (OptiEIA ELISA sets; BD Biosciences) as per the manufacturer’s protocol. The 50% inhibitory concentration (IC_50_) values were calculated by nonlinear regression method using GraphPad software (Prism5).

### Immunohistochemical analysis of tumor xenografts

The formalin fixed paraffin embedded sections of xenograft tumor tissue from control and treated groups were stained by immunohistochemistry for specific proteins. The slides were deparaffinized followed by antigen retrieval by incubating the slides in citrate based antigen unmasking solution (pH 6.0) (Vector Labs, CA, USA) for 30 minutes. Slides were then blocked for two hours with 1X blocking buffer (containing 2% FBS, 2% FCS, 5% goat serum, 5% donkey serum, 1%BSA and 0.1% triton-x) followed by incubation with primary antibody overnight at 4°C. Slides were incubated with secondary antibody conjugated with DyLight 549 fluorescent dye for 60 minutes at 37°C and mounted with Vectastain® Elite ABC reagent. All tumor tissues without primary antibody were used as negative control throughout the study. Images were captured with Nikon E600 microscope equipped with a DXM1200F digital camera (Nikon USA). Pixel intensities were measured using ImageJ software (http://rsb.info.nih.gov/ij/).

## Results

### *In vitro* activity of P276-00 on HNSCC cells

**Figure 1:**
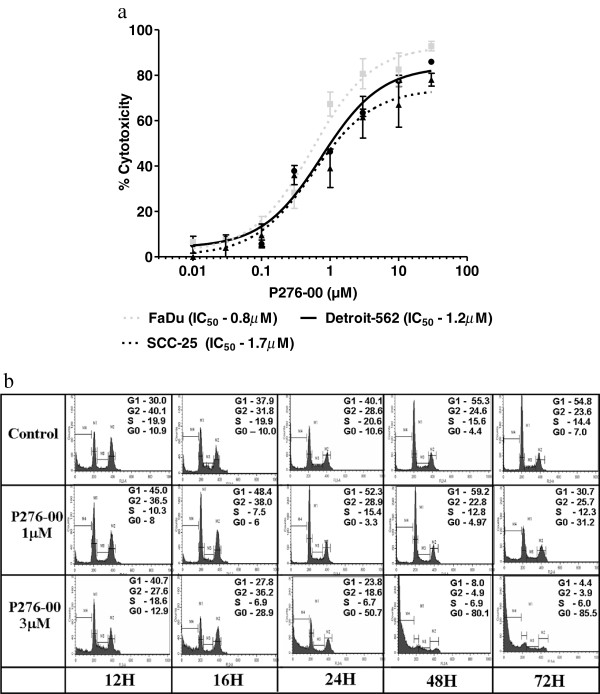
***In vitro *efficacy of P276-00 caused by G1 arrest in HNSCC cells (a) Anti-proliferative effect of P276-00 on HNSCC cells using CCK-8 assay.** Cells (Detroite-562, filled circles; SCC25, filled triangles, and FaDu, filled boxes) were treated to 5 different concentrations of P276-00 (range: 0.1-10 μM) and measured after 48 hours. Here data is expressed in % cytotoxicity mean ± S.E.M. (n = 3). The IC_50_ of each cell line was calculated. (**b**) Flow cytometric analysis of asynchronous population of FaDu cells exposed to either P276-00 (1 and 3 μM) or DMSO for different time points (12, 16, 24, 48 and 72 hours). Histograms of cellular DNA content obtained by flow cytometry have been represented. The (%) DNA content for each phase of the cell cycle is indicated, which was determined by ModFit analysis.

Anti-cancer properties of P276-00 were tested across multiple HNSCC cell lines (FaDu, Detroits-562, SCC-25) by measuring dehydrogenase based tetrazolium salt reduction produced by viable cells (Figure 1a). The results showed significant inhibition of cell viability for all three cell lines with IC_50_ value ranging from 0.8 to 1.7 μM depending on specific cell lines.

### P276-00 caused G1 arrest in *FaDu* cells

To determine the effect of P276-00 on specific cell cycle stages, cells exposed to two different concentrations of the drug were assayed for their cell cycle status at different time points (Figure 1b). It was observed that P276-00 induced cell arrest at G1 phase within 12 hours of drug exposure. The effect peaked after 24 hours lasting up to 48 hours. P276-00 significantly reduced cells from the S phase in a dose-dependent manner. Our data also showed a moderate effect of P276-00 on G2/M phase of the cell cycle. Moreover, at 3 μM, more than 50% of cells appeared in sub-G0 phase after 24 hours of treatment which increased up to ~85% by 72 hours. These results suggest drug induced cell cycle blockade in G1 phase followed by apoptosis, as indicated by the appearance of cells with sub-G0 DNA content.

### P276-00 targeted CCND1/pRB mediated E2F1 pathway causing G1 arrest in FaDu cells

**Figure 2:**
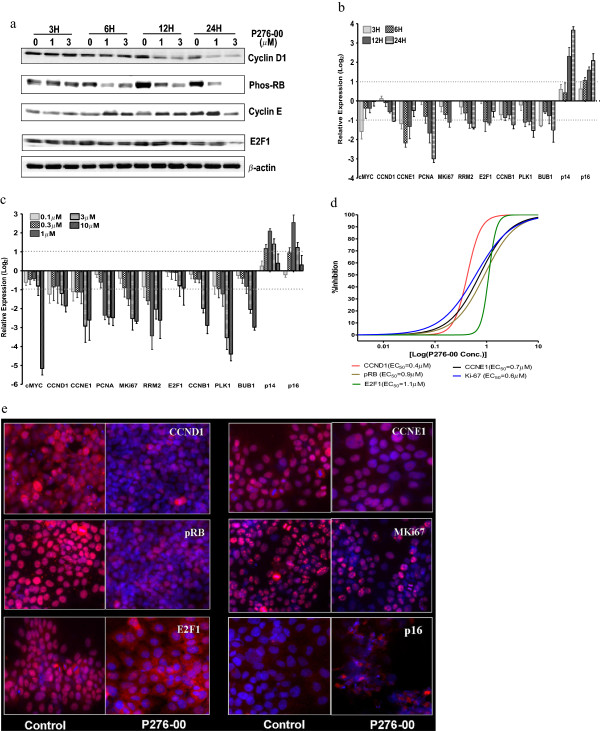
**P276-00 targeting CCND1/pRB mediated E2F1 pathway and causing G1 arrest in FaDu cells.** (**a**) Western blot analysis of CCND1, pRb, CCNE1 and E2F1 in FaDU cells treated with 1 and 3 μM P276-00 for 3, 6, 12 and 24 hours.(**b**-**c**) RTQPCR based transcript profiling of various downstream targets of E2F1 pathway. Data is represented on log2 scale as mean ± S.E.M. (n=3-5). (**b**) Dose response - FaDu cells were exposed to 5 different concentrations of P276-00 (range: 0.1-10 μM) for 12 hours in FaDu cells. (**c**) Time response- cells were exposed to 4 different time points (3, 6, 12 and 24 hours) with 1 μΜ of P276-00 (**d**-**e**) Automated fluorescence imaging based dose dependent protein profiling of CCND1/pRB mediated E2F1 pathway and its downstream targets**.** FaDu cells (1–2 × 10^4^ cells/ml) were treated with 8 different concentrations of P276-00 (range 0.03 to 10 μM) for 24 hours and observed using ArrayScan HCS reader after staining with respective antibody (**d**) Quantification of the automated fluorescence imaging data has been represented in sigmoidal dose response curve where compound concentration. is represented in log2 scale. One thousand cells were counted for each replicate well and the results were presented as an average ± S.E.M. (n = 3). EC_50_ values have been generated for all the 5 proteins. (**e**) Panel of representative images.

To establish the anti-proliferative properties of P276-00 via G1 arrest, we have investigated the status of CCND1/pRB mediated E2F1 signaling components in FaDu cells treated with or without P276-00 (Figure 2a). Treatment of FaDu cells with P276-00 showed a reduction in pRB and CCND1 protein levels as early as 6 hours and was maintained upto 24 hours. P276-00 also reduced E2F1 and CCNE1 protein expression by 24 hours (Figure 2a).

The inhibitory effect of P276-00 on E2F1 was further confirmed by measuring E2F1 target gene expression. P276-00 significantly inhibited twelve E2F1 targeted cell cycle genes (Additional file 1: Table S1) (>2 fold change, p < 0.05) as compared to untreated cells. Amongst these genes, CCND1, CCNE1, PCNA, KI67, cMYC, RRM2 and E2F1 expressions were consistently inhibited in a dose- and time-dependent manner upto 12 hours (only cMYC show a strong repression as early as in 3 hours) (Figure 2b, 2c). We observed a moderate inhibition of transcripts for CCNB1, PLK1 and BUB1 at all time points. In addition, transcript levels of negative cell cycle regulators including P16 and P14 were elevated in response to P276-00 exposure in a time-dependent manner. (Figure 2b, 2c)

Automated fluorescence imaging approach was used to measure the proteins that indicated molecular changes in response to P276-00 in FaDu cell lines. There was significant inhibition in expression of cell cycle proteins including CCND1, CCNE1, KI67, E2F1 and pRB. However, we found increased levels of P16 protein in response to P276-00 (Figure 2d, 2e). We determined the effective concentration (EC_50_) of P276-00 for specific target proteins (Figure 2d). Put together, these results suggest the inhibition order from low to high EC_50_ value as CCND1 (0.4 μM), KI67 (0.6 μM), CCNE1 (0.7 μM), pRB (0.9 μM) and E2F1 (1.1 μM). Furthermore, the slopes of dose–response plots (‘Hill’ slopes) were determined for the inhibition of all the proteins across 8 various concentrations. Two major observations emerged: The Hill slopes remains ~1 for CCNE1, KI67 and pRB with 8 different concentrations of the P276-00 suggesting that the proteins are inhibited in dose dependent manner. The steep Hill slopes with >3.4 value for CCND1 and E2F1 indicate an on-off response with treatment.

### P276-00 induced apoptosis through phosphorylation of P53 in FaDu cells

**Figure 3:**
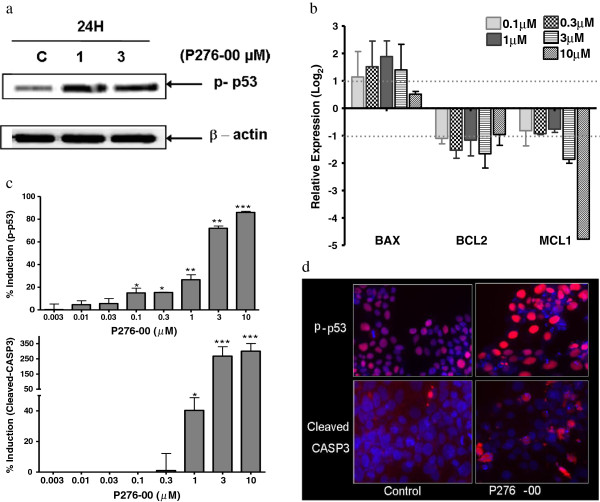
**Cytotoxic nature of P276-00 is due to phosphorylation of P53 in FaDu cells.** (**a**) Western blot analysis of phosphorylated-P53 in FaDu cells treated with 1 and 3 μM P276-00 for the 24 hours. (**b**) Dose dependent relative mRNA expression of downstream target of P53 after 24 hours of treatment. Data in histograms represented on log2 scale as mean ± S.E.M. (n = 3). (**c**-**d**) Dose dependent treatment of P276-00 (8 different concentrations -range 0.03 to 10 μM) for 24 hours has shown induction of P53 phosphorylation at Ser392 and cleavage of CASP3 in FaDu cells detected by Automated fluorescence imaging (HCS reader) (**c**) Graphical representation of the data (**d**) Representative images.

The extent of apoptosis is maintained by a well regulated balance between pro- and anti-apoptotic factors. In this regard, we demonstrated that P276-00 induced the expression of proapoptotic gene BAX at 12 hours of treatment and attenuated expression of anti-apoptotic genes BCL2 and MCL1 (Figure 3b). Cleaved CASP3, critical for induction of apoptosis, was also elevated in response to P276-00 in a dose-dependent manner (Figure 3c, 3d). To further probe the apoptotic mechanism of P276-00, we observed that P53 phosphorylation was significantly elevated *in vitro* in response to P276-00 (Figure 3a, 3c, 3d). This observation is consistent with an increase in BAX and BCL2 genes (Figure 3b), since the latter are P53 target genes as well.

### P276-00 inhibited growth signaling proteins and cytokines in FaDu cells

**Figure 4:**
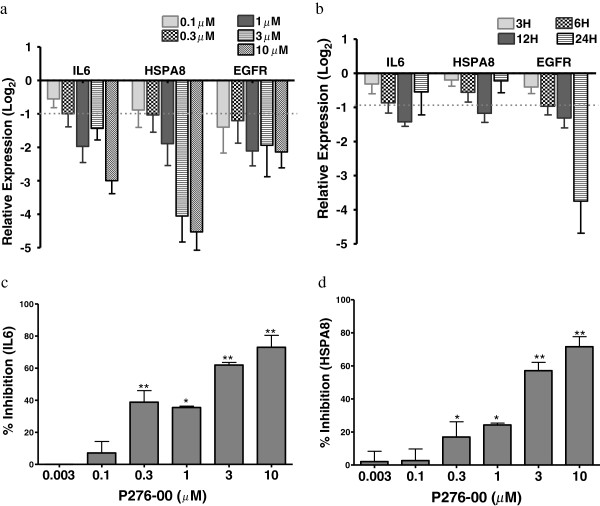
**P276-00 inhibit growth signaling proteins and cytokine in FaDu cells.** (**a**-**b**) P276-00 inhibits mRNA level of IL-6, EGFR and HSPA8 in FaDu cells. The relative expression level of these genes was measured in comparison with control sample (**a**) Time dependent response (**b**) dose response. (**c**-**d**) P276-00 potently suppresses *in vitro* production of IL-6 and HSPA8 in FaDu cells. (**c**) IL-6 (**d**) HSPA8. All values are average ± S.E.M. (n=3-5) * indicates p-val < = 0.05 as compared to DMSO pre-treated control cells.

We examined growth and immune signalling proteins such as EGFR, HSPA8 and cytokines IL-6 that are involved in the survival of tumor. There was a marked inhibition of HSPA8 and IL-6 genes at mRNA level in dose- and time-dependent manner (Figure 4a, 4b). In congruence with the gene expression, P276-00 significantly inhibited IL-6 and HSPA8 protein levels measured by ELISA with IC_50_ at 1.4 μM and 1.2 μM respectively (Figure 4c, 4d). EGFR gene expression was significantly reduced by 1 μM P276-00 in a time-dependent manner with maximal inhibition at 24 h (Figure 4b). Additional file 1: Figure S1 showed a marked dose-dependent inhibition of EGFR gene expression at 24 h with a not so robust dose–response effect at 12 h (Figure 4a).

### Effects of P276-00 *in vivo* on FaDu xenograft model

**Figure 5:**
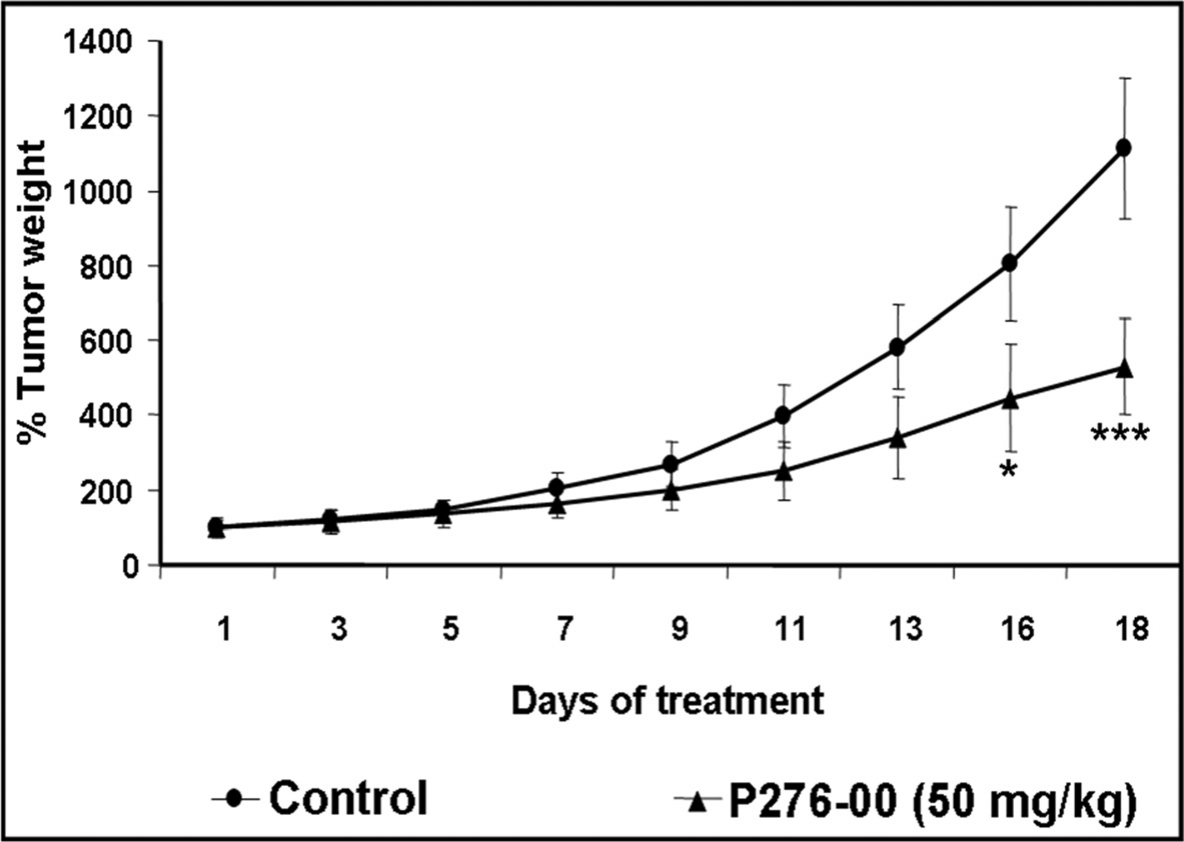
**Effects of P276-00 on FaDu xenograft.** Average tumor growth of HNSCC (FaDu) xenograft over a period of 18 day after randomization. Group treated with P276-00 (shaded square), control group (open square) (n = 6).

**Figure 6:**
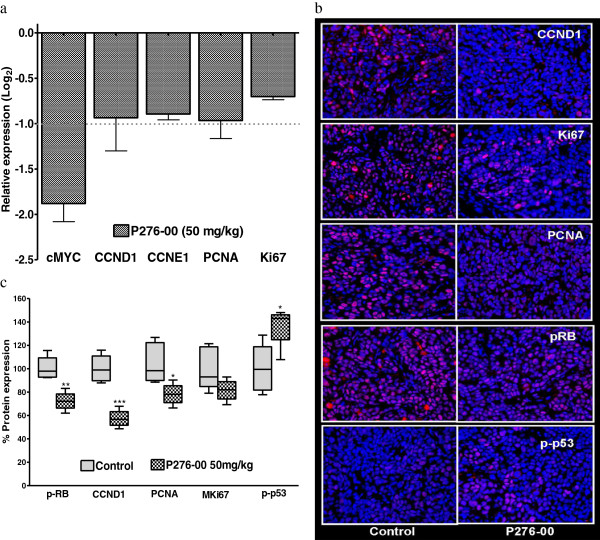
**Effects of P276-00 on CCND1/pRB mediated E2F1 pathway in FaDu xenograft model. a**) RTQPCR based mRNA profiling of E2F1 downstream targets using tumor from HNSCC (FaDu) xenograft model. Data is represented at log2 scale mean ± S.E.M. (n = 3–4). (**b**) Representative images from immunohistochemical analysis of various proteins in FaDu xenograft tumors- P276-00 treated mice (right) or vehicle (left), (**c**) graphical representation of immunohistological score.

Given the significant anti-proliferative and pro-apoptotic effects of P276-00 in HNSCC *in vitro*, we investigated the anti-tumor efficacy in a HNSCC tumor xenograft mouse model. P276-00 showed significant (p < 0.001) *in vivo* activity with a 48% tumor growth inhibition (TGI) on day 18 (Figure 5). Consistent with the *in vitro* evidence, P276-00 inhibited cell cycle gene expression in the tumor tissue including CCND1, CCNE1, cMYC, KI67 and PCNA (>1.8 fold inhibition or fold change in gene expression compared to control group Figure 6a). Immunohistochemical analysis of tumor tissue for molecular targets of cell cycle such as CCND1, CCNE1, pRB and PCNA demonstrated a significant decrease in expression level accompanied by a moderate decrease in KI67 in response to P276-00 treatment (Figure 6b, 6c). Similar to *in vitro* studies, FaDu xenograft tumors showed increased levels of phosphorylated P53 in response to P276-00 administration (Figure 6b, 6c).

## Discussion

P276-00, a novel CDK inhibitor, is currently under clinical investigation for HNSCC (http://clinicaltrials.gov/show/NCT00824343). In the present study, we have demonstrated anti-cancer properties of P276-00 against HNSCC using *in vitro* and *in vivo* tumor models. We observed significant inhibition of E2F1 and its targets via reduced RB phosphorylation by P276-00. We also demonstrated that P276-00 promoted apoptosis by phosphorylation of P53 resulting in increased BAX/BCL2 ratio and elevated levels of cleaved caspase-3. Furthermore, P276-00 inhibited expression of soluble proteins such as IL-6, EGFR and HSPA8 that promote tumor microenvironment.

The anti-cancer properties of flavones such as flavopiridol through CDK inhibition have been established in head and neck cancer [19]. P276-00, a flavone with a specific CDK1/4/9 inhibition activity, has been studied for anticancer effects in several other cancers but not HNSCC [15,16]. In the current study, we demonstrated the anti-cancer properties of P276-00 in HNSCC. Cell lines over expressing CCND1 viz., FaDu, Detroits-562 and SCC-25 were very sensitive to P276-00 as observed in the cytotoxicity assay, which is similar to previously reported evidence in other cell lines [15,16]. As FaDu cells have highest CCND1 expression and accordingly showed highest *in vitro* sensitivity to P276-00, we employed these cells for subsequent investigations *in vitro* and *in vivo*.

We observed that P276-00 resulted in 48% tumor growth inhibition (TGI) by day 18 post drug administration with minimal toxicity to animals as observed by the animal body weight loss (less than 10% of total body weight loss -data not shown) in the FaDu xenograft model. This TGI value is comparable to the reported data for anti-tumor activity (40% optimal TGI) [20]. Thus our *in vivo* study with FaDu xenograft model establishes the therapeutic validity of P276-00.

CDK inhibitors interfere with the cell cycle progression through inhibition of cell proliferation and induction of apoptosis. We have evaluated the status of P276–00 treated FaDu cells by flow cytometry and observed that P276-00 blocks cell cycle progression by G1 arrest. Our findings are in accordance with a prior report by Carlson and colleagues, who demonstrated that flavopiridol causes cell cytotoxicity by G1-S phase arrest in MDA-MB-468 breast carcinoma cells [21]. RB protein, a tumor suppressor that blocks E2F transcription factor, regulates transcription of E2F-responsive genes involved in cell cycle progression from G1 to S phase. It is well established that CDK4/CCND1 complex phosphorylates RB and thus releases E2F to initiate transcription [6-8] of genes such as cMYC, CCNE1, PCNA, KI67, RRM2 and E2F1 [22-26] which are crucial for the entry of cells into the S phase. On the contrary, P16 inhibits phosphorylation of RB protein and attenuates E2F1 activity. Loss of P16 expression has been observed in 55% of HNSCC tumors [4]. We observed a significant inhibition of CCND1 expression and RB phosphorylation followed by reduced transcription of E2F1 targeted genes in presence of P276-00 under both *in vitro* and *in vivo* conditions. The on-off dose-responses for CCND1 and E2F1 have important clinical implications suggesting that lower concentrations of P276-00 can be clinically used without noticeable loss in efficacy. These observations suggest that P276-00 mediates its anti-proliferative activity via inhibition of CCND1/pRB/E2F1 pathway leading to G1-S phase arrest. On the other hand, G2/M check point markers CCNB1, PLK1 and BUB1 [25,27] showed marginal inhibition by P276-00, which further compliments the moderate arrest of cells in G2/M phase in FACS analysis. Human ARF/INK4a locus encodes two cell cycle inhibitors, P16INK4a (P16) and P14ARF (P14), which are directly modulated by E2F1 [28,29]. P276-00 induced the expression of P16 and P14 in a time-dependent manner at lower dose levels. Low levels of P16 and P14 transcript observed with the higher concentration of P276-00 could be due to overall E2F1 mediated transcriptional repression by P276-00. The temporal relationship among abrogated levels of phosphorylated RB and E2F1 targets explains inhibition of G1 phase of the cell cycle via CCND1/pRB/E2F1 pathway.

Prolonged cell cycle arrest causes apoptosis [30]. FaDu cells are known to express mutant P53 with a point mutation on codon 248 [31]. Despite this point mutation, phosphorylation of P53 at Ser392 results in transactivation of P53 and oncogenic function of P53 mutant [32]. Activated (phosphorylated) P53 trans-represses anti-apoptotic BCL-2 [33,34] and over expresses pro-apoptotic BAX [33], which triggers the caspase cascade. Consistently, P276-00 treatment in FaDu cells showed increased P53 phosphorylation at Ser392 and cleaved CASP-3 expression along with reduced BCL2 to BAX ratio, which was associated with enhanced apoptosis (measured by FACS). The decrease in proapoptotic BAX mRNA expression at higher concentrations of P276-00 could be due to overall CDK9/CyclinT1-mediated repression of basal transcriptional machinery by P276-00 [17]. However, this needs additional validation.

Growth signaling proteins and cytokines, that promote cell growth, inflammation, and angiogenesis, play an important role in tumor microenvironment enhancing tumor proliferation, invasion, and metastasis [35]. Molecular pathogenesis of HNSCC is also driven by similar alterations in growth and immune signalling [12,36]. HNSCC tumors get nourished due to its microenvironment and are well supplied with soluble epidermal growth factor receptor (EGFR) and cytokine (IL-6), which play a critical role in tumor aggressiveness and their response to various therapies [12,36]. Higher levels of IL-6 and EGFR in HNSCC cancer patients’ serum are found to be associated with a higher second primary cancer (SPC) specific mortality and hence established as serum biomarkers for HNSCC [14]. Dose-dependent inhibition of EGFR and IL-6 by P276-00 supports its sensitivity against growth promotion of HNSCC. Additionally, HSPA8 is a member of the heat-shock protein 70 families and known to promote cancer cell growth [13]. HSPA8 is important for stability of CCND1 mRNA and also helps to form a complex with CDK4 [37]. Interestingly, we have observed significant inhibition of HSPA8 by P276-00 at mRNA and protein levels in dose-dependent manner. Based on these observations, we propose that the secretary levels of IL-6 and HSPA8 could be the potential efficacy biomarker for P276-00 treatment in the clinic.

## Conclusions

We conclude that P276-00 has potent anti-proliferative and anti-tumor effects in HNSCC *in vitro* and *in vivo*. The findings from our study provide mechanistic insights into the anti-cancer activity of P276-00 and its effect on CCND1/CDK4/P16/pRB/E2F signaling cascade in FaDu cells. Being the key mediators of cancer survival, pharmacological inhibition of E2F1 targets is an attractive option for therapeutic intervention against cancer. Therefore, P276-00 can serve as a promising candidate in HNSCC therapy, particularly for those lesions that over expresses CCND1 and are insensitive to conventional therapy.

## Competing interests

All the authors except Gopinath A Kumar are employees of Piramal Healthcare Ltd. Gopinath A Kumar, director of SBST department at VIT University, serves as thesis supervisor for Prabha B Mishra and contributed to the planning and reviewing of manuscript. The authors declare that they have no competing interest.

## Authors’ contributions

PM was involved in conceptualizing the study and manuscript writing after data compilation. She was responsible for all the data generation and the subsequent analysis. AL was responsible for supporting PM for cell culture and western blot analysis. KJ was involved in the supervision and coordination of all the animal studies. MR was responsible for conducting experiments to generate mouse xenograft models. AG provided guidance for the work. MP the group leader in the Biomarker Discovery group was responsible for supervising and coordinating the whole work and has critically reviewed the manuscript. All the authors read and approve the final manuscript.

## Supplementary Material

Additional file 1: Table S1 Candidate gene set for RTQ-PCR analysis. List of genes and their primer sequences used for the RTQ-PCR analysis. The primer sequences were designed using Primer-3. **Figure S1.** P276-00 inhibit EGFR transcript levels in FaDu cells. The relative expression level of EGFR genes was measured after 24 hours of treatment of various doses of P276-00 in comparison with control sample. All values are represented as an average of N=3 data point ±S.E.M. Click here for file
