# Supplementary material for: Molecular mechanisms of anti-tumor properties of P276-00 in head and neck squamous cell carcinoma
Source: J Transl Med. 2013 Feb 18;11:42. doi: 10.1186/1479-5876-11-42 (PMC3672051; doi:10.1186/1479-5876-11-42)
Supplement: Additional file 1: Table S1 — Candidate gene set for RTQ-PCR analysis. List of genes and their primer sequences used for the RTQ-PCR analysis. The primer sequences were designed using Primer-3. Figure S1. P276-00 inhibit EGFR transcript levels in FaDu cells. The relative expression level of EGFR genes was measured after 24 hours of treatment of various doses of P276-00 in comparison with control sample. All values are represented as an average of N=3 data point ±S.E.M. [file 1479-5876-11-42-S1.doc]

| **Gene Symbol** | **Forward** | **Reverse** |
| --- | --- | --- |
| BAX | AGTAACATGGAGCTGCAGAGGAT | GCTGCCACTCGGAAAAAGAC |
| BCL2 | CATGTGTGTGGAGAGCGTCAA | GCCGGTTCAGGTACTCAGTCA |
| BUB1 | CGGATTTTTGGAACAGGATG | ACTCTGACCCAGGTCAATCAG |
| CCNB1 | GCCAGAACCTGAGCCTGTTA | GGCTTGGAGAGGCAGTATCA |
| CCND1 | GGTCTGTGCATTTCTGGTTGC | GCTGGAAACATGCCGGTTAC |
| CCNE1 | CTCCAGGAAGAGGAAGGCAA | TCGATTTTGGCCATTTCTTCA |
| CDKN2A/P16 | CAACGCACCGAATAGTTACGG | AACTTCGTCCTCCAGAGTCGC |
| E2F1 | CGTGGACTCTTCGGAGAACT | CTCAGGGCACAGGAAAACAT |
| HSPA8 | CCCATCATCACCAAGCTGTA | AGCTCCACCACCAGGAAAT |
| IL6 | TCGGGTAGTGGAAAACCAGC | TTCCTGTTGGTGAAGCTAACGTT |
| MCL1 | GGACCTAGAAGGTGGCATCA | CCAAACCAGCTCCTACTCCA |
| MKI67 | CCATGTGCCTGAGATCAAGA | TGCACAGATTTGCTCTCCAA |
| cMYC | CGGGAACGAAAGAGAAGCTCTA | GGCGCTTGTGGAGAAGGAG |
| PCNA | AATTGCGGATATGGGACACT | TGCCTAAGATCCTTCTTCATCC |
| PLK1 | TTCTTCCAGGATCACACCAA | CGCTTCTCGTCGATGTAGGT |
| GAPDH | ATTCCACCCATGGCAAATTC | GATGGGATTTCCATTGATGACA |
| P14 | CATAGATGCCGCGGAAGGT | CCCGAGGTTTCTCAGAGCCT |

**Supplementary table 1:** **Candidate gene set for RTQ-PCR analysis**. List of genes and their primer sequences used for the RTQ-PCR analysis. The primer sequences were designed using Primer-3.

**Supplementary figure 1:** **P276-00 inhibit EGFR transcript levels in FaDu cells**

The relative expression level of EGFR genes was measured after 24 hours of treatment of various doses of P276-00 in comparison with control sample. All values are represented as an average of N=3 data point ±S.E.M.
